# Intravenous Bolus Phenylephrine and Intravenous Bolus Norepinephrine for Treatment of Maternal Hypotension in Spinal Anesthesia During Cesarean Section: A Prospective, Randomized, Comparative Study

**DOI:** 10.7759/cureus.103343

**Published:** 2026-02-10

**Authors:** Danish Jamal Ansari, Manoj K Chaurasiya, Manish Singh, Ravi Prakash, Dinesh Kaushal

**Affiliations:** 1 Department of Anaesthesiology, King George’s Medical University, Lucknow, IND

**Keywords:** cesarean section, intravenous bolus norepinephrine, intravenous bolus phenylephrine, intravenous vasopressor, maternal hypotension

## Abstract

Background

A fall in maternal blood pressure is frequently encountered following spinal anesthesia for cesarean delivery. This hemodynamic disturbance primarily results from reduced venous return, leading to a decline in cardiac output and/or systemic vascular resistance. The primary objective of this study was to estimate the efficacy and safety of norepinephrine and phenylephrine for the treatment of maternal post-spinal hypotension during cesarean section. The secondary objective was to compare the hemodynamics and neonatal APGAR scores between the two vasopressors.

Methodology

This prospective, randomized trial included 72 parturients scheduled for cesarean section. Before the administration of spinal anesthesia, participants were allocated using computer-generated randomization into the following two groups: norepinephrine (Group A) and phenylephrine (Group B). Group A received an intravenous bolus of norepinephrine 4 μg (4 μg/mL), while Group B was administered an intravenous bolus of phenylephrine 50 μg (50 μg/mL). Both study drugs were prepared by dilution with normal saline.

Results

The mean systolic blood pressure showed a decline in both groups for up to the initial two minutes and a gradual elevation later. A significantly greater decline in heart rate was noted in the phenylephrine group (Group B) compared with the norepinephrine group (Group A) at multiple time points during observation. In addition, patients receiving phenylephrine required fewer rescue bolus doses than those receiving norepinephrine, with the difference reaching high statistical significance (p < 0.0001). Both groups showed no statistically significant difference (p = 0.3938) in neonatal outcome, assessed by the one-minute APGAR score. The majority of neonates in both groups had APGAR scores in the moderate range (4-6), accounting for 88.89% in Group A and 94.44% in Group B. There was a statistically insignificant difference in APGAR score in both groups (p = 0.3139) at five minutes, with 100% and 97.22% of patients falling under the reassuring category of score 7-9 in Group A and Group B, respectively.

Conclusions

An intermittent bolus of norepinephrine is as effective as an intermittent bolus of phenylephrine for the treatment of maternal hypotension in spinal anesthesia during cesarean section. However, the incidence of heart rate reduction was much higher with phenylephrine than with norepinephrine. The number of bolus doses required was higher with norepinephrine than with phenylephrine.

## Introduction

Cesarean delivery is increasingly performed using spinal anesthesia, as this technique minimizes airway-related risks and limits fetal exposure to anesthetic agents compared with general anesthesia [1]. However, spinal anesthesia is frequently associated with maternal hypotension, which may manifest as nausea, vomiting, or dizziness and can compromise uteroplacental perfusion, potentially resulting in fetal hypoxia and acidosis. Prompt correction of hypotension using intravenous fluid therapy and/or vasopressor agents is therefore essential to prevent adverse maternal and neonatal outcomes [2]. Commonly employed vasopressors during cesarean section include ephedrine and phenylephrine, with norepinephrine being adopted more recently in clinical practice [2].

Each vasopressor used during cesarean delivery has distinct benefits and limitations. Ephedrine has been associated with undesirable effects such as maternal tachycardia and an increased risk of neonatal acidosis [2]. Because it is associated with a lower incidence of fetal acidosis than ephedrine, phenylephrine has emerged as the vasopressor of choice for spinal anesthesia-related hypotension in cesarean sections [3]. However, its predominant α-adrenergic activity may lead to a reduction in maternal heart rate and cardiac output, potentially affecting both maternal and fetal hemodynamics. Norepinephrine, a potent vasopressor with additional β-adrenergic effects, has recently gained attention as a viable alternative to phenylephrine for the treatment of hypotension following spinal anesthesia during cesarean delivery [4,5]. Owing to its mixed adrenergic action, norepinephrine is believed to preserve heart rate and cardiac output more effectively than phenylephrine [6]. Previous studies have suggested that a bolus dose of phenylephrine 100 μg is approximately equipotent to norepinephrine 8 μg [7].

Intermittent bolus administration of norepinephrine offers superior maternal cardiac output and neonatal safety, making it optimal for preeclamptic cesarean deliveries. Phenylephrine is effective for blood pressure control but may induce bradycardia, while the use of ephedrine is limited by its association with neonatal acidosis. Tailored vasopressor selection is thus essential for optimal outcomes.

The primary objective of the present study was to compare the efficacy and safety of bolus norepinephrine and phenylephrine in the treatment of maternal hypotension following spinal anesthesia for cesarean section. Secondary objectives included evaluation of maternal hemodynamic responses to these vasopressors and assessment of neonatal outcomes through comparison of APGAR scores between the two groups.

## Materials and methods

This prospective, randomized trial was conducted over a one-year period (October 27, 2021, to October 26, 2022) in the operating theaters of the Department of Obstetrics and Gynecology at King George’s Medical University, Lucknow, India. The study protocol was registered with the Clinical Trials Registry-India (CTRI/2022/09/045141). A total of 72 parturients were enrolled and allocated in equal numbers to the following two groups using computer-generated randomization: the norepinephrine group (Group A; n = 36) and the phenylephrine group (Group B; n = 36). Participants were randomly allocated to the study groups using a computer-generated randomization sequence with a 1:1 allocation ratio. Allocation concealment was ensured using sequentially numbered, sealed, opaque envelopes prepared by an independent researcher. The study was conducted in a double-blind manner, with both participants and outcome assessors blinded to group allocation.

Eligible participants included women with American Society of Anesthesiologists (ASA) physical status I or II, carrying a singleton term pregnancy, and scheduled for elective cesarean delivery under spinal anesthesia. Exclusion criteria comprised age below 18 years or above 45 years; height outside the range of 150-180 cm; body weight less than 50 kg or exceeding 100 kg; contraindications to spinal anesthesia; known hypersensitivity to the study medications; and the presence of medical or obstetric comorbidities such as preeclampsia, placenta previa, diabetes mellitus, hypertension, or cardiovascular disease. Patients who declined to provide consent were also excluded from the study.

The sample size was calculated using the following formula: $n = \frac{\left( \sigma^{2}_{1} + \sigma^{2}_{2} / \kappa \right)\left( Z_{1-\alpha/2} + Z_{1-\beta} \right)^{2}}{\bigtriangleup ^{2}}$ 

Where *n* denotes the sample size, σ₁ represents the standard deviation of the first group (0.04), and σ₂ represents the standard deviation of the second group (0.06). Δ indicates the expected difference in means (0.03), while κ denotes the allocation ratio between the groups. The value Z₁-α⁄2 corresponds to the two-sided Z value (1.64), and Z₁-β represents the Z value corresponding to the study power (0.84).

Study protocol

All participants were assessed by an anesthesiologist during the pre-anesthetic visit on the day before surgery. Standard fasting protocols were followed, and patients were advised to remain nil per oral for at least eight hours before the procedure. As part of aspiration prophylaxis, intravenous ranitidine 50 mg and metoclopramide 10 mg were administered. In the operating room, patients were positioned supine with a left lateral tilt, and routine monitoring, including non-invasive blood pressure measurement and pulse oximetry, was instituted. An 18-gauge intravenous cannula was secured in a forearm vein and connected to a three-way stopcock without prior fluid preload.

The primary outcome, blood pressure and heart rate, and the secondary outcome, APGAR score, were measured at different time periods. Baseline systolic and diastolic blood pressures (SBP and DBP) were calculated as the average of three consecutive readings taken at one-minute intervals, with less than 10% variation between measurements. The corresponding mean heart rate values were considered baseline heart rates. Subarachnoid block was performed in the sitting position using either a Quincke or Whitacre spinal needle at the L2-L3 or L3-L4 intervertebral space. After confirmation of free cerebrospinal fluid flow, 1.8 mL of 0.5% hyperbaric bupivacaine combined with 0.5 mL (25 μg) fentanyl was injected intrathecally. Simultaneously with intrathecal drug administration, Ringer’s lactate solution was infused at a dose of 10 mL/kg.

Following the establishment of the subarachnoid block, patients were placed in a supine position with a routine left lateral tilt and maintained in this position until delivery of the neonate. Adequacy of spinal anesthesia was confirmed by the achievement of a bilateral sensory block to the T4-T5 dermatome level, assessed using a pinprick method within 10 minutes of intrathecal drug administration. Failure of spinal anesthesia was defined as the inability to attain a T4 sensory level within the specified time frame, and such patients were excluded from further analysis.

Before spinal anesthesia, participants were allocated through computer-generated randomization into the two study groups (Group A and Group B). An intravenous bolus of norepinephrine 4 μg (4 μg/mL) was given to patients in Group A, whereas those in Group B were administered phenylephrine 50 μg (50 μg/mL). All study medications were prepared by dilution with normal saline.

Non-invasive blood pressure and heart rate were monitored every two minutes during the initial 10 minutes after spinal anesthesia, followed by five-minute intervals until the end of the procedure. Hypotension was defined as a decrease in SBP of at least 20% from baseline or an absolute value below 100 mmHg, while hypertension was considered a ≥20% rise from baseline systolic pressure. Episodes of bradycardia (heart rate <60 beats/minute), nausea, and vomiting were also recorded. Hypotensive episodes were managed using repeat bolus doses of the assigned vasopressor (norepinephrine 4 μg or phenylephrine 50 μg). In cases where hypotension persisted despite two consecutive doses, vasopressin was administered as rescue therapy. Bradycardia with a heart rate below 55 beats/minute was treated with intravenous atropine 0.5 mg.

Key intraoperative time points, including initiation of spinal anesthesia, surgical incision, delivery of the neonate, and any technical difficulties encountered during surgery, were recorded. We documented APGAR scores after birth at one and five minutes, along with the neonatal birth weight.

Statistical analysis

All collected data were compiled in Microsoft Excel (Microsoft Corp., Redmond, WA, USA) and analyzed using SPSS software, version 26 (IBM Corp., Armonk, NY, USA). Mean and standard deviation were calculated for continuous variables. The chi-square test was used for categorical data. The independent t-test and repeated-measure analysis of variance were applied for data analysis. We considered p-values <0.05 to be significant.

Ethical considerations

Ethical approval (reference number: VI-PGTSC-IIA/P49) was obtained from the Institutional Ethics Committee of King George’s Medical University, Lucknow, India. We also obtained informed written consent from all the patients before the start of the study.

## Results

**Figure 1:**
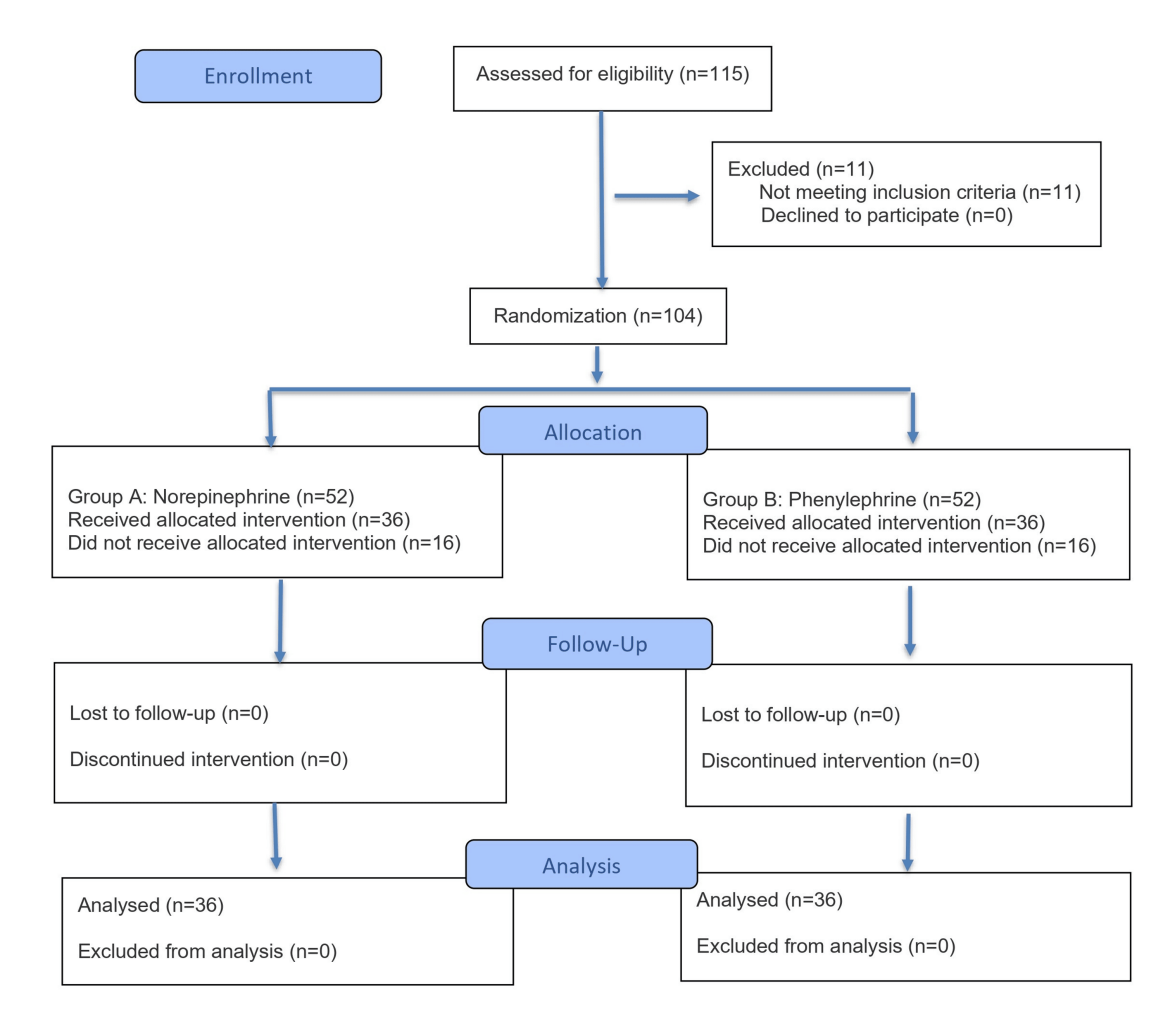
CONSORT flow diagram. n: number of patients; CONSORT: Consolidated Standards of Reporting Trials

The present study enrolled 115 patients. In total, 11 patients did not meet the inclusion criteria. Randomization was done for 104 patients, with 52 patients in each group. A total of 16 patients in each group did not develop hypotension. Finally, 72 patients were randomly allocated into two equal groups: Group A (n = 36, norepinephrine) and Group B (n = 36, phenylephrine (Figure 1). Participants assigned to Group A were administered an intravenous bolus of norepinephrine at a concentration of 4 μg/mL, whereas those in Group B received an intravenous bolus of phenylephrine at a concentration of 50 μg/mL.

In Group A, most participants were between 25 and 29 years of age (19 patients, 52.78%), followed by those aged 30-35 years (13 patients, 36.11%). In contrast, Group B predominantly included patients aged 30-35 years (17 patients, 47.22%), with the 25-29 year age group comprising 15 (41.67%) patients. Both groups showed no statistically significant difference (p = 0.6053) in terms of age distribution, as shown in Table 1.

**Table 1:** Age-wise distribution of enrolled patients. The chi-square test was used between the two groups. N: number of patients, %: percentage of patients

Age (years)	Group A (N = 36)		Group-B (N = 36)		P-value
	N	%	N	%	
20–24	4	11.11%	4	11.11%	$\chi$^2 ^= 1.004, p = 0.6053
25–29	19	52.78%	15	41.67%	
30–35	13	36.11%	17	47.22%	
Grand total	36	100.00%	36	100.00%	

Baseline anthropometric characteristics were assessed for both cohorts. The average height recorded in Group B (158.42 ± 5.25 cm) was slightly greater than that observed in Group A (156.64 ± 5.25 cm). Similarly, the mean body weight was marginally higher in Group B (59.03 ± 4.25 kg) compared with Group A (57.36 ± 4.70 kg). Body mass index (BMI) values followed the same trend, with Group B showing a marginally higher mean BMI (23.54 ± 0.87 kg/m²) than Group A (23.38 ± 1.02 kg/m²). None of these variations was statistically significant (Table 2).

**Table 2:** Anthropometric profile of the total enrolled patients. The independent t-test was used between the two groups. N: number of patients; BMI: body mass index; SD: standard deviation

Parameter	Group A (N = 36)	Group B (N = 36)	P-value
	Mean ± SD	Mean ± SD	
Height (cm)	156.64 ± 5.25	158.42 ± 5.17	t = 1.449, p = 0.1517
Weight (kg)	57.36 ± 4.70	59.03 ± 4.25	t = 1.581, p = 0.1183
BMI	23.38 ± 1.02	23.54 ± 0.87	t = 0.7161, p = 0.4763

Heart rate measurements were recorded at predefined intervals for both groups. Table 3 presents pairwise comparisons of heart rate (beats/minute) between baseline and successive time points up to 70 minutes. Across most time intervals, the mean differences in heart rate from baseline were small, and the 95% confidence intervals (CIs) consistently crossed zero, indicating no statistically significant change over time (all p ≥ 0.59). In the first comparison (baseline vs. two minutes), one column demonstrated a statistically significant reduction in heart rate (mean difference = −5.56 beats/minute; 95% CI = −9.78 to −1.33; p = 0.001), whereas the corresponding comparison in the other column was not significant (p = 1.000). Beyond this early time point, no significant differences were observed at 4-70 minutes, suggesting stabilization of heart rate with minimal fluctuation around baseline values. Overall, these findings indicate that heart rate remained largely stable throughout the observation period, except for a transient early decrease observed at two minutes in one comparison. The lack of sustained or progressive changes over subsequent time points suggests no clinically meaningful alteration in heart rate over time under the studied conditions.

**Table 3:** Heart rate profiling of the total enrolled patients. The repeated-measures analysis of variance was applied. CI: confidence interval; Sig.: significance value

Heart rate (beats/minute)		Mean difference	95% CI		Sig.	Mean difference	95% CI		Sig.
			Lower	Upper			Lower	Upper	
Baseline	2 minutes	-0.17	-3.92	3.59	1.000	-5.56	-9.78	-1.33	0.001
	4 minutes	-1.19	-4.95	2.56	1.000	1.56	-2.67	5.78	0.998
	6 minutes	0.58	-3.17	4.34	1.000	1.86	-2.37	6.09	0.987
	8 minutes	0.61	-3.14	4.37	1.000	0.75	-3.48	4.98	1.000
	10 minutes	1.56	-2.20	5.31	0.992	0.72	-3.51	4.95	1.000
	15 minutes	0.94	-2.81	4.70	1.000	0.39	-3.84	4.62	1.000
	20 minutes	0.33	-3.42	4.09	1.000	-0.14	-4.37	4.09	1.000
	25 minutes	-0.14	-3.89	3.62	1.000	-0.39	-4.62	3.84	1.000
	30 minutes	0.00	-3.76	3.76	1.000	-0.56	-4.78	3.67	1.000
	35 minutes	0.31	-3.45	4.06	1.000	-1.00	-5.23	3.23	1.000
	40 minutes	0.50	-3.26	4.26	1.000	-1.56	-5.78	2.67	0.998
	45 minutes	-0.11	-3.87	3.64	1.000	-2.06	-6.28	2.17	0.966
	50 minutes	0.72	-3.03	4.48	1.000	-2.92	-7.15	1.31	0.595
	55 minutes	0.58	-3.17	4.34	1.000	-2.17	-6.40	2.06	0.945
	60 minutes	1.97	-1.78	5.73	0.923	-1.90	-6.16	2.36	0.985
	65 minutes	0.62	-4.86	6.11	1.000	-1.42	-6.15	3.31	1.000
	70 minutes	-	-	-	-	-2.06	-7.71	3.59	0.999

SBP trends were also monitored over time. Table 4 presents pairwise comparisons of SBP (mmHg) between baseline and subsequent time points up to 70 minutes. A marked and statistically significant increase in SBP from baseline was observed during the early phase of observation. At two minutes, SBP increased by approximately 21-22 mmHg in both comparisons (p < 0.001), with narrow 95% CIs that did not cross zero. This elevated SBP persisted at 4, 6, 8, 10, and 15 minutes, with mean differences ranging from about 7 to 17 mmHg, all remaining highly significant (p < 0.001). At 20 and 25 minutes, the increase in SBP was smaller but still statistically significant in both comparisons (mean difference = ~4-6 mmHg; p ≤ 0.032). From 30 minutes onward, no statistically significant differences in SBP compared with baseline were detected, as the mean differences were minimal and the 95% CIs consistently included zero (all p ≥ 0.35). These findings indicate that SBP rose significantly immediately after baseline, reached a peak in the early time points, and then progressively declined toward baseline values, with normalization occurring by approximately 30 minutes. Overall, the results suggest a transient and time-dependent increase in SBP without sustained elevation over the later observation period.

**Table 4:** Systolic blood pressure profiling of the total enrolled patients. The repeated-measures analysis of variance was applied. SBP: systolic blood pressure; CI: confidence interval; Sig.: significance value

SBP (mmHg)		Mean difference	95% CI		Sig.	Mean difference	95% CI		Sig.
			Upper	Lower			Upper	Lower	
Baseline	2 minutes	20.72	16.09	25.36	0.000	21.64	17.66	25.62	0.000
	4 minutes	13.86	9.23	18.50	0.000	16.78	12.80	20.76	0.000
	6 minutes	15.89	11.25	20.52	0.000	12.14	8.16	16.12	0.000
	8 minutes	10.75	6.11	15.39	0.000	10.28	6.30	14.26	0.000
	10 minutes	10.22	5.59	14.86	0.000	8.47	4.49	12.45	0.000
	15 minutes	8.61	3.98	13.25	0.000	6.94	2.96	10.93	0.000
	20 minutes	5.44	0.81	10.08	0.006	5.94	1.96	9.93	0.000
	25 minutes	5.06	0.42	9.69	0.017	4.14	0.16	8.12	0.032
	30 minutes	3.64	-1.00	8.27	0.348	2.75	-1.23	6.73	0.593
	35 minutes	3.08	-1.55	7.72	0.661	1.92	-2.07	5.90	0.969
	40 minutes	2.94	-1.69	7.58	0.736	1.08	-2.90	5.07	1.000
	45 minutes	2.08	-2.55	6.72	0.984	0.61	-3.37	4.59	1.000
	50 minutes	1.31	-3.33	5.94	1.000	0.42	-3.57	4.40	1.000
	55 minutes	0.31	-4.33	4.94	1.000	0.11	-3.87	4.09	1.000
	60 minutes	0.67	-3.97	5.30	1.000	0.64	-3.34	4.62	1.000
	65 minutes	0.33	-4.30	4.97	1.000	0.70	-3.34	4.74	1.000
	70 minutes	1.09	-4.40	6.57	1.000	1.03	-4.79	6.85	1.000

Regarding bolus dose requirements, half of the patients in Group A (18 patients, 50.00%) required two bolus doses, while 10 (27.78%) patients required only one dose. Conversely, the majority of participants in Group B (29 patients, 80.56%) were adequately managed with a single bolus, and the remaining patients (7 patients, 19.44%) required two doses. Patients receiving phenylephrine required a significantly lower number of bolus doses compared with those in the norepinephrine group, with the difference reaching strong statistical significance (p < 0.0001) (Table 5).

**Table 5:** Number of bolus doses for the total enrolled patients. The chi-square test was used between the two groups. N: number of patients; %: percentage of patients; *: significant

Number of bolus doses	Group A (N = 36)		Group B (N = 36]		P-value
	N	%	N	%	
1	10	27.78%	29	80.56%	$\chi$^2 ^= 2.154, p = 0.0078*
2	18	50.00%	7	19.44%	
3	8	22.22%	0	0.00%	
Grand total	36	100.00%	36	100.00%	

Analysis of gender distribution showed a slight predominance of females in Group A (19 patients, 52.78%) compared with males (17 patients, 47.22%). In Group B, males were more frequently represented (20 patients, 55.56%) than females (16 patients, 44.44%). These differences were not statistically significant (p = 0.4793) (Table 6).

**Table 6:** Neonatal gender status for total enrolled patients The chi-square test was used between the two groups. N: number of patients; %: percentage of patients

Neonatal gender	Group A (N = 36)		Group B (N = 36)		P-value
	N	%	N	%	
Female	19	52.78%	16	44.44%	$\chi$^2^=0.5004, p = 0.4793
Male	17	47.22%	20	55.56%	
Grand total	36	100.00%	36	100.00%	

Neonatal outcomes were assessed using APGAR scores. At one minute, most neonates in Group A (32 cases, 88.89%) achieved scores within the moderate range (4-6). A similar pattern was observed in Group B, where 34 (94.44%) neonates fell within the same category. Table 7 shows that there was no statistically significant difference (p = 0.3938) between the groups.

**Table 7:** APGAR score at one minute for the total enrolled patients. The chi-square test was used between the two groups. N: number of patients; %: percentage of patients

APGAR score at 1 minute	Group A (N = 36)		Group B (N = 36)		P-value
	N	%	N	%	
4–6 (moderate)	32	88.89%	34	94.44%	$\chi$^2 ^=0.7273, p = 0.3938
7–8 (reassuring)	4	11.11%	2	5.56%	
Grand total	36	100.00%	36	100.00%	

At five minutes, all neonates in Group A (36 cases, 100%) attained reassuring APGAR scores ranging from 7 to 9. In Group B, 35 (97.22%) neonates achieved comparable scores. The observed outcomes were similar across both groups, and statistical analysis confirmed the absence of a significant difference (p = 0.3139) (Table 8).

**Table 8:** APGAR score at five minutes for the total enrolled patients. The chi-square test was used between the two groups. N: number of patients; %: percentage of patients

APGAR score at five minutes	Group A (N = 36)		Group B (N = 36)		P-value
	N	%	N	%	
6 (moderate)	0	0.00%	1	2.78%	$\chi$^2 ^= 1.014, p = 0.3139
7-9 (reassuring)	36	100.00%	35	97.22%	
Grand total	36	100.00%	36	100.00%	

## Discussion

This study evaluated the effectiveness of intermittent bolus norepinephrine versus phenylephrine for managing maternal hypotension after spinal anesthesia. According to the results of the current study, both groups of pregnant patients who received either phenylephrine or norepinephrine for post-spinal hypotension experienced the same primary outcome. The norepinephrine group required more boluses than the phenylephrine group to treat maternal hypotension. Phenylephrine causes the heart rate to drop more frequently than norepinephrine does. Maternal hypotension must be treated as early as possible to reduce the chance of fetal acidosis. Several vasopressors are advised for managing maternal hypotension caused by spinal hypotension due to their ability to constrict blood vessels. Spinal hypotension may be managed using vasopressors administered as either intermittent boluses or continuous intravenous infusions, with the latter offering improved blood pressure stability and reduced clinician intervention [8]. When infusion pumps are scarce, intermittent bolus administration offers a practical alternative to continuous vasopressor infusion during cesarean section. Thus, even though phenylephrine infusions have been demonstrated to be superior to boluses, phenylephrine boluses are still often utilized in many centers to treat spinal-induced hypotension. Norepinephrine has the additional benefit of being more affordable than phenylephrine. The administration of norepinephrine in the peripheral vein raised some questions. However, when it was administered through a peripheral vein, there were no symptoms of ischemic problems in the limbs [9,10].

Multiple studies have explored the equipotent dosing of norepinephrine and phenylephrine for managing spinal anesthesia-induced maternal hypotension. The ED90 for an intermittent bolus of norepinephrine has been reported as 6 µg [11]. Based on the findings of Ngan Kee [7], we used a bolus dose of 4 µg norepinephrine, which is approximately equipotent to 50 µg of phenylephrine. Similar results have been reported by Mohta et al. [12], who found norepinephrine to be roughly 11 times more potent than phenylephrine, and by Sharkey et al. [13], who demonstrated more stable hemodynamic control with norepinephrine (6 µg) compared with phenylephrine (100 µg) due to less fluctuation in heart rate.

In our study, we observed that 4 µg of norepinephrine is as effective as 50 µg of phenylephrine in treating maternal hypotension while performing cesarean section using spinal anesthesia. Despite a marginally lower requirement for bolus dose in the phenylephrine group, maternal and neonatal outcomes were similar across the two groups. These findings align with previous reports indicating that intermittent boluses of norepinephrine effectively manage spinal hypotension without adversely affecting neonatal outcomes [14-16]. Wang et al. [15] reported similar efficacy between norepinephrine and phenylephrine, with improved maternal and neonatal safety in parturients with preeclampsia, while Xu et al. [16] noted comparable APGAR scores at one and five minutes and a reduced incidence of side effects such as bradycardia and intraoperative nausea and vomiting with norepinephrine.

Moreover, prior studies [17,18] have demonstrated that norepinephrine and phenylephrine boluses achieve similar control of maternal blood pressure, with no significant differences in secondary outcomes such as nausea, vomiting, or neonatal APGAR scores. Similarly, Goel et al. [19] found that norepinephrine infusion was as effective as phenylephrine in maintaining SBP, with a lower incidence of bradycardia, while Mohta et al. [20] reported comparable efficacy for bolus doses of 5 µg norepinephrine and 100 µg phenylephrine in elective cesarean sections. Consistent with these studies, our results indicate that 4 µg norepinephrine and 50 µg phenylephrine are equally effective for treating maternal hypotension, with similar maternal and neonatal outcomes; however, phenylephrine was associated with a greater reduction in heart rate. Sundararajan et al. (2022) reported that intermittent bolus administration of norepinephrine is an effective strategy for the management of spinal anesthesia-induced hypotension during cesarean delivery. Their findings further demonstrated that, when used to maintain maternal blood pressure during spinal or combined spinal-epidural anesthesia, norepinephrine did not adversely affect neonatal outcomes compared with phenylephrine [21].

Limitations of the study

While the study has notable strengths, it is important to recognize its limitations. The sample of 72 participants, although adequate for preliminary analysis, was relatively modest for a clinically important comparison, and larger studies with greater statistical power are warranted. Multicentric trials with larger sample sizes may provide more precise and reliable estimates of efficacy and safety. In addition, the prospective, longitudinal, randomized design lacked a separate control group, which may limit the robustness of causal inferences. As this research was conducted in a single tertiary center, the findings may not be generalizable to wider populations or different healthcare environments. Consequently, extrapolation of these results to a larger population should be undertaken with caution.

Recommendations

Future research should focus on more extensive investigations addressing similar objectives while minimizing potential confounding factors. Future multicenter investigations with larger sample sizes are warranted to improve the robustness, accuracy, and external validity of the study findings. Additionally, well-designed longitudinal studies are warranted to evaluate and compare the maternal and fetal outcomes associated with intravenous bolus administration of phenylephrine versus norepinephrine for the treatment of maternal hypotension associated with spinal anesthesia.

## Conclusions

Norepinephrine given as intermittent boluses is equally effective as phenylephrine in treating maternal hypotension under spinal anesthesia for cesarean section. However, the incidence of heart rate reduction was much higher with phenylephrine than with norepinephrine. The number of bolus doses required was higher in norepinephrine than in phenylephrine. No notable differences were observed in maternal or neonatal outcomes between the groups.
